# Prevalence and associated factors of depression and anxiety among patients with cancer seeking treatment at the Butaro Cancer Center of Excellence in Rwanda

**DOI:** 10.3389/fpubh.2023.972360

**Published:** 2023-02-16

**Authors:** Samuel Habimana, Emmanuel Biracyaza, Tharcisse Mpunga, Epaphrodite Nsabimana, Florence Kayitesi, Pascal Nzamwita, Stefan Jansen

**Affiliations:** ^1^Department of Social work and Social Ecology, School of Behavioral Health, Loma Linda University, Loma Linda, CA, United States; ^2^Rwanda Resilience and Grounding Organization, Kigali, Rwanda; ^3^Faculty of Medicine, School of Rehabilitation, Université de Montréal, Montréal, QC, Canada; ^4^Rwanda Ministry of Health, Government of Rwanda, Kigali, Rwanda; ^5^Hope and Homes for Children, Kigali, Rwanda; ^6^Acquired Immunodeficiency Syndrome Health Care Foundation, Kigali, Rwanda; ^7^Center for Research and Innovation, College of Medicine and Health Sciences, University of Rwanda, Kigali, Rwanda

**Keywords:** cancer, depression, anxiety, cancer patient, oncology, patients

## Abstract

**Background:**

Depression and anxiety are psychological and physiological disturbances persisting in cancer patients with high prevalence worldwide, particularly in low- and middle-income countries, due to complexities of determinants of health including biological, individual, socio-cultural, and treatment-related characteristics. Although depression and anxiety have an enormous impact on adherence, length of stay at the hospital, quality of life, and treatment outcomes, studies on psychiatric disorders remain limited. Thus, this study determined the prevalence and factors of depression and anxiety among patients with cancer in Rwanda.

**Methods:**

A cross-sectional study was conducted among 425 patients with cancer from the Butaro Cancer Center of Excellence. We administered socio-demographic questionnaires and psychometric instruments. Bivariate logistic regressions were computed to identify significant factors to be exported into the multivariate logistic models. Then, odds ratios and their 95% confidence intervals were applied, and statistical significance at *p* < 0.05 were considered to confirm significant associations.

**Results:**

The prevalence of depression and anxiety was 42.6 and 40.9%, respectively. Patients with cancer initiated to chemotherapy had a greater likelihood of being depressed [AOR = 2.06; 95% CI (1.11–3.79)] than those initiated to chemotherapy and counseling. Breast cancer was significantly associated with a greater risk of depression [AOR = 2.07, 95% CI (1.01–4.22)] than Hodgikins's Lymphoma cancer. Furthermore, patients with depression had greater odds of developing anxiety [AOR = 1.76, 95% CI (1.01–3.05)] than those with no depression. Those suffering from depression were almost two times more likely to experience anxiety [AOR = 1.76; 95% CI (1.01–3.05)] than their counterparts.

**Conclusion:**

Our results revealed that depressive and anxious symptomatology is a health threat in clinical settings that requires enhancement of clinical monitoring and prioritization of mental health in cancer health facilities. Designing biopsychosocial interventions to address associated factors needs special attention to promote the health and wellbeing of patients with cancer.

## Background

Currently, cancer is the second largest cause of death worldwide and one of the most pressing public health concerns. Unfortunately, the predictions indicate that this public health burden will increase in the next two decades (1). Breast, lung, colorectal, prostate, and skin cancers are the most prevalent types of cancer. Depending on their diagnosis, patients with cancer undergo specialized treatment options such as surgery, radiation, and chemotherapy (2). When an individual is diagnosed with cancer, they experience emotional reaction problems, anxiety anger and depression, and other related mental health problems (3). In 2019, there were 23.6 million new global cancer cases (17.2 million), 10.0 million cancer deaths, and an estimated 250 million disability-adjusted life years lost (DALYs) due to cancer; since 2010, these represent increases of 26.3, 20.9, and 16.0%, respectively, which requires appropriate strategies to combat with this burden. Since 2010, the absolute cancer burden has increased in all Socio-demographic Index (SDI) quintiles, but the percentage increases have been greatest in the low and low-middle SDI quintiles (1, 3, 4). According to targets 3 and 4 of the Sustainable Development Goals (SDG) of the United Nations (UN), there is an estimate of the reduction of one-third of premature mortality from non-communicable diseases (NCDs) through the appropriate prevention and treatment and promotion of mental health and wellbeing. The health interventions will have a significant contribution to the reduction of the burden of cancer. This will occur if all countries of the world step up their efforts to lower the burden of NCDs including cancer (5, 6).

Depression and anxiety are psychosocial burdens that decimate the quality of life of patients with cancer. This psychiatric disorder has been reported as one of the threats to the health of patients with cancer due to its increase in morbidity and mortality. Depression and anxiety are the most common and incapacitating neuropsychiatric disorders among patients with cancer (2). Earlier studies stated that depression and anxiety disorders are psychological and physiological disorders characterized by physical, emotional, and behavioral symptomatology, which require psychological intervention in order to promote the mental health and wellbeing of the patients (7). Depression is defined as a mood disorder characterized by five or more symptoms occurring in the first 2 weeks of onset (8, 9). Its symptoms are insomnia, suicidal ideation, fatigue, eating and sleeping disturbances, loss of weight, psychomotor problems, and rumination about the loss or loss of interest. Depression is highly prevalent in developed and developing countries (10), and as a result, it is the fourth leading cause of disabilities worldwide (11, 12). Indeed, depression indicates its association with disability-adjusted life years (DALYs) and impacts quality-adjusted life expectancy (QALYs) in patients with cancer and the general population (13, 14), significantly in low- and middle-income countries (LMICs) (15, 16). These disabilities and conditions were aggravated by the COVID-19 pandemic, threatened adherence, and led to social withdrawal and social isolation in the community, while anxiety disorder is characterized by a sensation of tension, concerned thoughts, and physical changes such as elevated blood pressure (17, 18).

Psychiatric disorders, particularly depressive, and anxiety disorders, are important concerns in patients with cancer. These disorders have diverse impacts such as hampering treatment compliance in patients with cancer and worsening the treatment responses. It is through that lens, early interventions and appropriate monitoring are essential to promote the health of patients with cancer (19, 20). In addition, depression and anxiety may hinder cancer treatment, recovery, quality of life, and survival in clinical practices (20).

They can be significant barriers to management and control, adherence to the treatments, length of stay at the hospital, and ultimately survival rate (21, 22). Preceding studies on epidemiology reported that patients with cancer are more likely to experience depressive and anxiety disorders than the general population (19, 20, 23). Thus, more than one in four patients with cancer experience depression or anxiety, which requires psychotherapies and medical treatments (11). Although cancer survivors are expected to rise exponentially from about 16 million in 2016 to about 27 million in 2040, cancer is taking the highest toll (24, 25). Astoundingly, more deaths due to cancer in developing countries are probably due to compromised treatment, including limited psychosocial intervention (24, 26). Regarding the prevalence, other studies documented that depression in patients with cancer is three times higher than that in the general population (20, 27). The incidence rate of depression among patients with cancer was 71.8% (28, 29). The prevalence of depression and anxiety was significantly higher in adult patients with cancer compared with people without cancer, where depression was 54.9% in patients with cancer and 17.5% in people without cancer, and anxiety was 49.69% in patients with cancer vs. 18.37% in people without cancer (30, 31).

Furthermore, a higher proportion of anxiety and depressive disorders are associated with various factors including individual factors (family history, personal psychiatric history, personality traits, and demographic characteristics), interpersonal and social factors (loneliness, fear of losing life, social isolation, stigma, low socio-economic outcomes, lack of social or family supports, and living in stressful life events), and health-related complications or treatments including types of treatments, types of cancer, and side effects of medications (32). This complexity of symptomatology of psychiatric disorders is very common because two-thirds of cancer patients with depression also develop anxiety disorders. Depression and anxiety mutilate quality of life (QOL) among patients with cancer (33, 34). They may also cause loss of sensation, low libido, social problems, sticky saliva, dry mouth, and weight loss (34, 35).

Concerning the types of cancer, the rate of depression varies and differs from one sample to another (36, 37). Patients with oropharyngeal cancer are more likely to experience depression (22–57%) than those with pancreatic cancer (33–50%). Patients with breast cancer increased from 1.5 to 46%, whereas patients with lung cancer increased from 11 to 44% (23, 34). The prevalence of depression ranges proportionally for the different forms of cancer, ranging from 8 to 19% for lymphoid cancer to 13–25% for colon cancer (38, 39).

Patients with liver, stomach, and colon malignancies had a marked rise in depression and anxiety problems as a result of the rising number of cancer survivors (40). On the other hand, depression is highly prevalent before patients with cancer have initiated the treatments; however, a combination of chemotherapy and psychotherapy seems to be a better way to treat cancer and decrease mental disorders among these people. This indicates that the impact of treatments increases the quality of life of patients with cancer (38). Apart from the impact of treatments, depression is highly prevalent among hospitalized patients than in out-patients (28). The stress caused by a cancer diagnosis and its treatment can disrupt existing health behaviors or exacerbate unhealthy behaviors (41, 42). These stresses may also lead to anxiety and depression disorders (40, 43).

Even though, depression and anxiety are known to be common in patients with cancer and their prevalence is higher than in the general population, as documented in previous studies, these mental disorders are often neglected (44). Based on the evidence mentioned earlier, assessment for psychiatric disorders among patients with cancer is substantial for identifying which patients need psychological interventions and further assessment and thus, subsequent mental healthcare services. However, very little has been known about the prevalence of depression and anxiety disorders and their associated factors in oncological practices. As a result, this study was conducted to determine the prevalence of depression and anxiety disorders and their associated factors among patients with cancer seeking treatment at the Butaro Cancer Center of Excellence (BCCE) in Rwanda.

## Methods

### Study settings

The study area is the BCCE located within Butaro District Hospital in the Northern Province, Burera district, Butaro Sector of Rwanda. This health facility was founded in 2011 and designed by the Mass Design Group in cooperation with the Rwanda Ministry of Health and Partners in Health. It is the first rural-based specialty referral facility to provide cancer care and pathology-based diagnostic services in East Africa. The hospital has different services, including internal medicine, pediatrics, obstetrics and gynecology (OB/GYN), surgery, emergency and neonatology units, HIV/AIDS care, integrated non-communicable diseases clinics, and mental health (45, 46). The mental health department also has an out and inpatients clinic. In this department, all health facilities in the Burera district refer their patients with psychiatric and psychological diseases to be treated at Butaro hospital, the only facility with qualified psychotherapists. The current center provides healthcare interventions among outpatients and inpatients of all ages and genders to promote their health.

### Study design

A cross-sectional study was conducted among patients with cancer seeking healthcare services at BCCE of the Butaro District Hospital in Rwanda.

### Participants and procedures

The data collection was performed from December 2019 to February 2020 among the patients with cancer who came to seek healthcare interventions at the BCCE. Data were collected from the inpatient and outpatient settings using the convenience sampling technique. Patients with any type of cancer, willing to participate, and provide consent to participate in this survey formed the study population. This study's inclusion criteria were patients with cancer seeking health interventions at BCCE for more than 6 months, with an age range of 18–66 years, and whose living conditions were good enough to participate in the study. The study excluded patients with cancer who had medical conditions that could not allow them to respond to the research questions and patients with cancer who had a psychiatric history. It also excluded patients aged below 18 years or above 66 years and those with the apparent cognitive deficit or physical distress that did not permit them to participate. The healthcare providers and team of researchers of this study identified and assessed eligible participants.

### Sample size

The sample size was calculated using the proportion of depression reported in prior studies that stated 40% of depression among patients with one or more communicable diseases nationwide (47). The formula of Cochran ($n_{0}=\frac{z^{2}*p\left(1-p\right)}{e^{2}}$) was employed to compute the sample size. The parameters from this formula are “stands” for the minimal sample size, “***z***” for the confidence intervals with the value of “95% that is equivalent to 1.96,” “***p***” for the updated proportion of depression (=0.4), and “***e***” stands for the margin error (e = 0.05). Using this formula ($n=\frac{1.96^{2}*0.4(1-0.4)}{0.05^{2}}=368.8≃369$) and adding the extra 13% to the sample size for increasing the sample size, a total of 425 patients with cancer were enrolled in this study.

### Data collection and materials

The demographic and medical background data of patients with cancer were collected from the inpatients and outpatients within the hospital setting. Data collection was conducted by the data collectors, who were provided training for 5 days to help them become familiar with psychometric tools and research purposes—the information about the diseases extracted from patients' medical files. The data collectors were fluent in the respondents' mother tongue (Kinyarwanda). Data collectors were also psychologists, psychiatrists, and sociologists who are trained to support emotional regulation if it happens during data collection.

#### Socio-demographic questionnaire and medical characteristics

It comprises age, residence, province, education, marital status, sex, social or Ubudehe category, and employment. Medical conditions compromise the variables such as the history of depression in family relatives, duration of cancer disease, patients under treatment, type of cancer, types of therapies being provided, and patients with another chronic disease.

#### Beck depression inventory

BDI is the psychometric instrument used to assess the severity of depression (40). This instrument validates the assessment of the severity of depressive symptoms that has high sensitivity and predictive value. It covers 21 items with a Likert scale from 0 to 3 scales, with a maximum achievable score of 63. Its results show that a score <14 shows minimal symptoms of depression, a score from 14 to 19 indicates mild depressive symptoms, a score from 20 to 28 indicates moderate depressive symptoms, and a score ≥29 indicates severe depressive symptoms (41). The cutoff for the BDI-II is 17. This study found that BDI-II had an adequate Cronbach's Alpha (BDI-II, α = 0.912), which was also documented in previous studies conducted in the same medical setting (40, 42).

#### State-Trait Anxiety Inventory-Trait

STAI-T is a psychometric instrument used to measure the severity of anxiety (42). STAI-T is one of the parts of the State-Trait Anxiety Inventory (STAI) with high test-retest reliability, internal consistency, and concurrent validity with another anxiety questionnaire (43). Previous studies conveyed that the scores of STAI-T vary from a minimum score of 20 to a maximum score of 80 on both the STAI-T and STAI-S subscales. STAI scores are commonly classified as “no or low anxiety” (20–36), “moderate anxiety,” and “high anxiety” (44). Therefore, the cutoff is 37, and its internal consistency coefficients have ranged from 0.86 to 0.95. In this study, we used the STAI-T, whose scores range from 20 to 80 and show a greater level of anxiety (43, 45). It measures the rate of several anxiety-related symptoms regarding how the individuals feel. Items are scored from 1 (rarely) to 4 (almost always) on a four-point Likert-type scale, with the total score ranging from 20 to 80. This instrument presented good reliability and validity in patients with cancer (46). As this instrument had good psychometric properties in schools in Rwanda (47), our study also reported that the STAI-I presented an adequate internal consistency (Alpha of Cronbach, α = 0.956).

### Data analysis

Data were entered into Microsoft Excel for processing and then imported into Statistical Package of Social Sciences (SPSS) software version 25 for statistical analyses. The summative model was used to determine the consistency of the tests of Cronbach's Alpha of BDI-II. Descriptive statistics were carried out for the socio-demographic characteristics (SDC) and the disease conditions of the study participants, including sex, age, education, orphanage, residence, and marital status. These demographic data and disease conditions were associated with the prevalence and severity of the depressive disorder. The associations among predictable factors such as socio-demographic and disease conditions and depressive disorder were measured. Bivariate logistic analysis was computed to determine associations between independent factors and depression and anxiety. Then, the Pearson correlations between the STAI-T and BDI were applied based on the relationship among depression, the satisfaction of the patients with the services provided, anxiety as a comorbidity of depression, and the patient's trust in their medical care providers. We sued the independent variables with 95% confidence intervals and 5% for statistical significance were employed in the bivariate analyses and multivariate logistic regression models based on odds ratios to indicate significant factors of anxiety and depressive disorders among patients with cancer.

### Ethics

The Institution Review Board (IRB) of the College of Medicine and Health Sciences at the University of Rwanda issued, following the regulations and standards of the Helsinki Declaration (48), permission to conduct this study with the reference number (Ref. No: 136/UR-CMHS/SPH/2019). The participants who agreed to participate in this study were explained the purpose and objectives and were provided informed consent. Then, before data collection, the enrolled patients with cancer provided signed informed consent. For the patients from palliative care departments, they provided the assent to participate while their caregivers provided the written consent forms. Confidentiality and voluntariness were ensured. All data were kept anonymously.

## Results

### Socio-demographic characteristics of patients with cancer at BCCOE

Table 1 shows various types of cancers with their prevalence; breast cancer is 44%, headache, cervical, and brain cancer are 15.8%, chronic myeloid leukemia is 8.2%, Hodgkin's Lymphoma is 6.8%, Kaposi cancer is 4.2%, chronic lymphocytes leukemia is 3.5%, gastric cancer is 3.1%, GTN cancer is 2.4%, and other types of cancer are 12%. Most patients with cancer (79.3%) seeking healthcare were women. Regarding educational status, most (44%) patients with cancer studied in primary schools. Results showed that many cases were more than 49 years of age, including 132 (31.1%) and 110 (19.5%) patients aged 50–59 years and more than 59 years, respectively. Most of the patients with cancer [146 (34.4%) and 181 (42.6%)] were from the second- and third-social categories designed by the Rwandan government using standardized measurements. Concerning the therapies provided to the patients, most of the patients (*n* = 145, 34.1%) were provided mixed chemotherapy and psychotherapy. More than half (25.9%) of patients with cancer received only chemotherapy, while 23.1% were offered other surgical and radiology interventions. Only 67 cases (15.8%) had a family history of depression (Table 1).

**Table 1:** Description characteristics of the patients with cancer (*n* = 425).

**Demographic characteristics**	**Number**	**Percent**
**Residence**		
Rural	316	74.40
Urban	109	25.60
**Sex**		
Female	337	79.30
Male	88	20.70
**Awareness of cancer disease**		
No	7	1.60
Yes	418	98.40
**Province**		
North	104	24.50
South	100	23.50
East	78	18.40
West	100	23.50
Kigali	43	10.10
**Duration with cancer**		
<1 year	138	32.50
1–5 years	262	61.60
>5 years	25	5.90
**Presence of depression in first-degree relatives (like a close**		
**family members)**		
No	358	84.20
Yes	67	15.80
**Patients under treatment**		
No	55	12.90
Yes	370	87.10
**Type of therapies being provided**		
Chemotherapy	110	25.90
Counseling	7	1.60
Chemotherapy, counseling, and palliative care	65	15.30
Chemotherapy and counseling	145	34.10
Other types of therapies	98	23.10
**Patients age in years**		
18–29 years	47	11.10
30–39 years	53	12.50
40–49 years	110	25.90
50–59 years	132	31.10
>59 years	83	19.50
**Highest achieved education**		
Illiterate	166	39.10
Primary	187	44.00
Secondary	59	13.90
University	6	1.40
VTC	7	1.60
**Marital status**		
Single	51	12.00
Married	263	61.90
Separated/divorced	111	26.10
**Ubudehe category (social category)**		
Category 1: those living in abject poverty	95	22.30
Category 2: very poor	181	42.60
Category 3: poor	146	34.40
Category 4: resourceful poor	3	0.70
**Employment**		
Employed	15	3.50
Unemployed	376	88.50
Student	8	1.90
Self-employed	26	6.10
**Types of cancer**		
Hodgkin's Lymphoma	29	6.80
Gastric cancer	13	3.10
Breast cancer	187	44.00
Headache/cervical/brain	67	15.80
Chronic lymphocytic leukemia	15	3.50
Chronic myeloid leukemia	35	8.20
Kaposi cancer	18	4.20
GTN	10	2.40
Other types of cancer	51	12.00
**Patients with other chronic diseases**		
No	344	80.90
Yes	81	19.10

GTN, gestational trophoblastic neoplasia; VTC, vocational training center. Category I (Those living in abject poverty): It includes those who lived by begging or who are dependent on others; Category 2 (very poor): It is for those with poor housing poor diet, depending on others, and not having own land or livestock; Category 3 (Poor): It is for malnourished people, who owned a small portion of land, low production capacity and could not afford secondary school education for their kids; Category 4 (resourceful poor): This is for those who owned some land, cattle, a bicycle, and production capacity. They afford secondary school education for their kids and face fewer difficulties accessing healthcare.

### Prevalence of depression and anxiety among patients with cancer undergoing inpatient treatment at BCCOE

Concerning the prevalence of anxiety and depression among patients with cancer, the results indicated that the depression was higher and near the cutoff of depression using BDI-II (*M* = 16.3, *SD* = 9.8). A high prevalence of depression (42.6%) and anxiety disease using traits of anxiety (40.9%) was found among patients with cancer. The results indicated that the level of trait anxiety was high with a mean of 36.8 (*SD* = 12.6) (Table 2).

**Table 2:** Prevalence of depression and anxiety among patients with cancer (*n* = 425).

**Psychometric analysis**	**Number**	**Prevalence**
BDI mean ± SD (range)	16.3 ± 9.8 (0–50)	
**Depression (17 is the cut-off points)**		
No	244	57.40
Yes	181	42.60
**STAI-T (cut-off is 37)**		
No	251	59.10
Yes	174	40.90
STAI-T mean ±SD (range)	36.8 ± 12.6 (20–64)	

SD, standard deviation; BDI, Beck Depression Inventory; STAI-T, State-Trait Anxiety Inventory Trait Version.

The results reported significant correlations between the psychometric measures used in this study, such as a significant correlation between STAI-T and BDI-II (*r* = 0.08, *p* < 0.01).

### Factors associated with depression among the patients undergoing healthcare services

Among many variables, bivariate logistic regression analyses indicated that the significant factors of depression were gender, age, type of cancer, history of depression in the family, type of treatments, and the traits of anxiety. These significant variables were exported into multivariate logistic regression models and as a result types of treatments, types of cancer, gender, and trait of anxiety become significant factors of depression. Hence, the male patients were less likely to be depressive [AOR = 0.37; 95% CI (0.14–0.98)] than female patients. Cancer patients with family relatives diagnosed with depression had almost six times more likely to be depressed [AOR = 5.8; 95% CI (3.1–10.7)] than their counterparts. Those with the traits of anxiety disorders were almost three times more likely to be depressive [AOR = 2.9, 95% (2.23–3.8)] than those with no traits of anxiety disorders. Furthermore, patients with cancer under chemotherapy only had greater odds of developing depressive disorders than those under both chemotherapy and psychological therapies [AOR = 2.06, 95% CI (1.11–3.79)]. Providing a combination of palliative care, chemotherapy, and counseling was associated with an increase in the odds of being depressive [AOR = 1.62; 95% CI (0.95–2.75)] compared to those under chemotherapy and counseling. In addition, the participants under other treatments (radiology or chirurgical) were almost two times more likely to be depressed [AOR = 1.55; 95% CI (0.9–2.62)] than the patients with cancer under chemotherapy and counseling. The diagnoses of breast cancer [AOR = 1.01; 95% CI (0.59–4.22)] and cervical (headache or brain) cancer [AOR = 1.60; 95% CI (1.02–5.14)] increased the risk for depression than Hodgkin's Lymphoma cancer. But those with gastric cancer [AOR = 0.31; 95% CI (0.08–1.41)], chronic lymphocytic leukemia [AOR = 0.71; 95% CI (0.45–1.09)], chronic myeloid leukemia [AOR = 0.45; 95% CI (0.14–1.43)], Kaposi [AOR = 0.48; 95% CI (0.14–1.64)], GTN [AOR = 0.70; 95% CI (0.35–1.04)], and other types of cancer [AOR = 0.60; 95% CI (0.48–1.06)] were associated with a decrease of depression compared to those with Hodgkin's Lymphoma (Table 3).

**Table 3:** Bivariate and multivariate logistic regression model analyses for the risk factors for depression.

**Characteristics**	**Bivariate logistic regression analysis**		**Multivariate logistic regression models**	
	**COR**	**95% CI**	**AOR**	**95% CI**
**Residence**				
Rural	1			
Urban	0.92	0.60–1.43		
**Province**				
North	1			
South	0.82	0.4–1.67		
East	0.75	0.37–1.54		
West	0.60	0.28–1.27		
Kigali	0.56	0.27–1.15		
**Knowing the disease**				
No	1			
Yes	0.22	0.03–1.85		
**Depression in other relatives**				
No	1			
Yes	2.10	1.19–3.71^*^	5.80	3.1–10.70^***^
**Type of cancer**				
Hodgkin's Lymphoma	1			
Gastric	0.27	0.06–1.17	0.31	0.08–1.41^*^
Breast	1.40	1.07–1.92	1.01	0.59–4.22
Headache/cervical/brain	2.90	2.20–3.80	1.60	1.02–5.41
Chronic lymphocytic leukemia	0.38	0.10–1.40	0.71	0.45–1.09^**^
Chronic myeloid leukemia	0.64	0.14–3.04	0.45	0.14–1.43^**^
Kaposi	0.45	0.12–1.78	0.48	0.14–1.64^***^
Other	0.35	0.08–1.60	0.60	0.35–1.04^**^
GTN	0.44	0.11–1.72	0.70	0.48–1.06^*^
**Under treatments**				
No	1			
Yes	1.60	0.91–2.82		
**Type of treatments**				
Chemotherapy and counseling	1		1	
Others (radiology, surgical)	1.29	0.78–2.13^*^	1.55	0.90–2.62^*^
Chemotherapy, counseling, and palliative care	1.56	0.93–2.62^*^	1.62	0.95–2.75^*^
Chemotherapy	1.93	1.07–3.48^*^	2.06	1.11–3.79^**^
**Duration of cancer disease**				
<1 year	1			
1–5 years	1.41	0.57–3.49		
>5 years	1.74	0.73–4.18		
**Other chronic diseases**				
No	1			
Yes	1.25	0.76–2.05		
**Age**				
18–29 years	1		1	
30–39 years	0.6	0.29–1.24^**^	0.64	0.30–1.36
40–49 years	0.5	0.24–1.01^**^	0.50	0.24–1.02
50–59 years	0.59	0.33–1.05^**^	0.62	0.34–1.12
>59 years	0.63	0.36–1.1^**^	0.68	0.38–1.20
**Gender**				
Female	1			
Male	0.39	0.36–0.41^*^	0.37	0.14–0.98^*^
**The highest education level achieved**				
Illiterate	1			
Primary	2.22	0.42–11.7		
Secondary	1.46	0.28–7.74		
University	2.26	0.41–12.6		
VTC	5	0.47–53		
**Marital status**				
Single	1			
Married	0.79	0.41–1.55		
Separated/ divorced/widowed	0.79	0.51–1.24		
**Ubudehe (social category) category**				
Category 1: those living in abject poverty	1			
Category 2: very poor	0.28	0.02–3.19		
Category 3: poor	0.36	0.03–4.10		
Category 4: resourceful poor	0.45	0.04–5.10		
**Employment status**				
Employed	1			
Unemployed	0.87	0.25–3.12		
Student	0.73	0.33–1.62		
Self-employed	0.33	0.06–1.97		
**STAI-T (cut-off is 37)**				
No	1		1	
Yes	1.77	1.20–2.61^**^	2.90	2.23–3.80^***^

^*^p < 0.05; ^**^p < 0.01; ^***^p < 0.001; STAI-T, State-Trait Anxiety Inventory Trait Version.

### Factors associated with anxiety disorders among patients with cancer undergoing treatments

Among diverse variables, bivariate logistic regression analyses reported that types of therapies, residence, history of depression in the family, having another chronic disease, marital status, and depression among patients with cancer were significant factors of anxiety traits. These essential and significant factors were analyzed relative to traits of anxiety disorders using the stepwise forward model of multivariate logistic regression models and then found three risk factors of anxiety. Therefore, the results demonstrated that patients with cancer diagnosed with depressive disorders were almost two times more likely to experience anxiety disorder [OR = 1.76, 95% CI (1.01–3.05)] than those diagnosed with depression. Cancer patients with other NCDs were almost three times more likely to be anxious [AOR = 2.54, 95% CI (0.66–9.8)] than patients with no NCDs. In addition, being under chemotherapy only [AOR = 1.99, 95% CI (1.12–3.53)] and combined surgery and radiology [AOR = 2.27, 95% CI (1.3–3.98)] was significantly associated with an increase of the risk for anxiety than being under chemotherapy combined with counseling. Interestingly, we discovered comorbidity of depression and anxiety disorders among patients with cancer in medical settings where depressed patients with cancer were almost two times to develop anxiety [AOR = 1.76, 95% CI (1.01–3.05)] when compared to their counterparts (Table 4).

**Table 4:** Bivariate and multivariate logistic regression models for the factors of anxiety.

**Characteristics**	**Bivariate logistic regressions**		**Multivariate logistic regression models**	
	**COR**	**95% CI**	**AOR**	**95% CI**
**Residence**				
Rural	1		1	
Urban	0.65	0.40–1.04^***^	1.41	0.84–2.37
**Province**				
Northern	1			
Southern	0.69	0.33–1.44		
Eastern	1.14	0.54–2.42		
Western	1.36	0.61–3.03		
Kigali city	0.74	0.35–1.55		
**Knowing the disease**				
No	1			
Yes	3.55	0.42–29.80		
**Depression in other relatives**				
No	1		1	
Yes	7.65	4.17–14^***^	1.66	0.89–3.07
**Type of cancer**				
Hodgkin's Lymphoma	1			
Gastric	1.29	0.31–5.27		
Breast	2.78	0.48–16.03		
Headache/cervical/ brain	1.84	0.54–6.28		
Chronic lymphocytic leukemia	1.25	0.35–4.49		
Chronic myeloid leukemia	0.74	0.16–3.39		
Kaposi	1.56	0.41–5.95		
Other	0.51	0.12–2.25		
GTN	1.39	0.37–5.21		
**Under treatments**				
No	1			
Yes	0.93	0.52–1.67		
**Type of treatments**				
Chemotherapy and counseling	1		1	
Others (surgery, radiology)	0.38	0.23–0.64^**^	2.27	1.30–3.98^**^
Chemotherapy, counseling, and palliative care	1.86	0.91–3.80^**^	0.66	0.31–1.41^*^
Chemotherapy	0.56	0.33–0.95^***^	1.99	1.12–3.53^**^
**Duration of cancer disease**				
<1 year	1			
1–5 years	0.65	0.31–1.2		
>5 years	0.47	0.18–1.22		
**Other chronic diseases**				
No	1		1	
Yes	2.58	0.60–11.15^**^	2.54	0.66–9.80^**^
**Age**				
18–29 years	1			
30–39 years	0.70	0.33–1.46		
40–49 years	0.96	0.47–1.97		
50–59 years	0.87	0.48–1.57		
>59 years	1.00	0.56–1.79		
**Gender**				
Female	1			
Male	1.58	0.98–2.54		
**Education**				
Illiterate	1			
Primary/VTC	0.62	0.13–2.85		
Secondary	0.54	0.12–2.47		
University	0.70	0.14–3.41		
**Marital status**				
Single	1		1	
Married	0.61	0.30–1.22^*^	1.78	0.83–3.85
Separated/divorced/ widowed	0.62	0.38–1.01	1.31	0.77–2.23
**Employment status**				
Employed	1			
Unemployed	1.75	0.48–6.35		
Student	2.10	0.94–4.63		
Self-employed	7.00	0.74–66.60		
**Depression**				
No	1			
Yes	1.63	1.10–2.41^*^	1.76	1.01–3.05^*^

^*^p <0.05; ^**^p < 0.01; ^***^p < 0.001; STAI-T, State-Trait Anxiety Inventory Trait Version.

## Discussion

To the best of our knowledge, depression and anxiety are mental disorders that have an impact on the health of patients with cancer, making them important in oncological settings. Despite their importance, little is known about their existence in patients with cancer in Rwanda. This study, hence, aimed to determine the prevalence of depression and anxiety and their associated factors among patients with cancer seeking healthcare services at the national center for patients with cancer in Rwanda. The results showed that 42.6 and 40.9% of patients with cancer had depressive and anxiety disorders, respectively, following the previous study that reported that depression and anxiety disorders are comorbid to cancer (49, 50). These results align with other studies that reported that patients with cancer developed a high prevalence of depression and anxiety (35). Yet, the prevalence of depression among patients with cancer is higher in many countries worldwide, including some countries in SSA, such as Uganda, Kenya, and Ethiopia (28, 51, 52). The prevalence of anxiety and depression reported in this study is less than the prevalence among patients with cancer from a referral hospital in Rwanda (36, 53). Our results reported that the prevalence of depression is greater than the prevalence of anxiety which is contradicted by the study conducted in Uganda confirming a higher prevalence of anxiety than depression in patients with cancer (52). Furthermore, our results are in disagreement with the other study in a Rwandan referral hospital which documented a higher prevalence of depression (67.7%) than anxiety disorder (52.1%) among patients with cancer (53).

Depression and anxiety had significant associations with the type of treatment. A high frequency was observed in patients who obtained chemotherapy as a single treatment. This was because patients under treatment for a long time are detached from their social supports and become stigmatized which might increase their level of vulnerability to depression. These results are not supported by the prior studies (54). In agreement with the earlier study on the causes of depression and anxiety disorders in SSA (55), our study reported that history of depression among the family members, types of cancer, gender of the patients, and traits of anxiety are some of the major factors contributing to depression. The patient with cancer whose family member was diagnosed with depression is our study's most vital associated risk factor of depressive disorder. More, the patients under a combination of chemotherapy and psychological therapies were less likely to be depressive compared to the patients with cancer being provided with other therapies. These results follow earlier studies (56, 57). However, only a few studies detected a relationship between depression and a history of depression among family members of patients with cancer.

The previous studies on cancer samples stated the positive impact of psychotherapies and counseling among patients with cancer in preventing a high risk of mental disorders and improving the quality of health (31, 58). Our findings proved that patients under psychotherapy or counseling were less likely to experience depression or anxiety. In agreement with the previous studies that indicated that types of cancer are the risk factor for depression and anxiety (35, 57), this study revealed that the type of cancer is the risk factor for depression. In contrast, breast cancer increases the odds of developing depressive disorders. Indeed, patients with cervical, headache, or brain cancer were highly exposed to depressive disorders. In contrast with the prior studies (59), the results of this study revealed that the type of cancer was not the risk factor for anxiety disorder.

Our results confirmed comorbidity between anxiety and depression among patients with cancer, where depressed patients were more likely to develop anxiety disorders than those without depression and vice versa. These findings contradicted other studies that reported anxiety as a risk factor for depression, and depression as a factor for anxiety disorder (19, 33). Several studies have investigated whether depression and anxiety are comorbid in patients with cancer (60). However, very few studies have detected anxiety symptoms as an associated factor for the comorbidity of depressive disorder in patients with cancer (61). Interestingly, male patients were at a lower risk to be depressed than female patients. Although these results are collaborating with the previous studies (43, 59), our findings could also be explained by a smaller number of male patients enrolled in our study compared to female patients.

Patients with cancer who were depressed were at greater risk to be anxious than their counterparts, which is supported by previous studies (50, 57). As a result, those with breast cancer, headache, or brain cancer were at a higher risk of depression than those with Hodgkin's Lymphoma. Furthermore, these associated characteristics did not increase the odds of being anxious except for the type of treatment. These results controvert the prior studies (36, 37). Surprisingly, being under chemotherapy only and combined surgery and radiology was significantly associated with an increase in the risk for anxiety than being under chemotherapy combined with counseling. We discovered comorbidity of depression and anxiety disorders among patients with cancer in medical settings. A recent study confirmed this epidemiological burden's diagnoses, health complications, and chronicity among patients with cancer (52, 55). Regarding the health complications, having other NCDs increased the risk for anxiety.

This study had several strengths. First, the study used standardized psychometric measures that were valid and reliable among the patients on target. Second, as the study was conducted among patients with cancer from the only National Center of Excellence of patients with cancer, where all Rwandan hospitals transfer their patients with cancer, the results are generalized to all patients with cancer in Rwanda under health interventions. Third, the sample size used was large. They have increased our results' precision, validity, and reliability. A large sample size increased our power to predict associations among depression, anxiety, and other associated variables based on the previous literature.

Although there were several strengths, numerous limitations were encountered. First, the study population consisted of patients with cancer enduring curative and palliative care, yet their psychological or emotional state is likely to differ significantly. Since all research participants were patients with cancer, researchers cannot compare some situations to the general population, such as type of residence, employment status, and marital status. Second, the study was limited to not measuring the patients' clinical presentations, which could cause over-rating or incorrect screening methods for anxiety and depression. Third, anxiety and depressive symptoms were assessed using self-report methods, which can never be equivalent to a well-structured clinical assessment and diagnosis. Fourth, the study was limited to the study design since some patients with cancer quickly elicit more severe symptoms and functioning impairments than others. Thus, cohort study designs for specific cancer diagnoses and longitudinal studies are better suited to provide adequate mental health outcomes and predictors of depression and anxiety among patients with cancer.

## Conclusion

There is a considerably high prevalence of depression and anxiety among patients with cancer. These mental disorders may create additional health concerns during the treatments and make cancer more challenging in terms of management and control and its complications. Types of medications and therapies, history of depression in the family, and types of cancer were the foremost factors in depression. Decision makers are recommended to design cancer management clinical guidelines to consider early assessment and management of depression and anxiety and continuously monitor it throughout treatment. Healthcare providers in primary and tertiary facilities need to recognize and screen for anxiety and depression among patients with cancer and provide appropriate psychological therapies. In addition, our results seem to make a timely contribution to the researchers and healthcare providers working in Rwanda, a low-income country, and other developed countries. To bring insight into the burden of mental disorders in this group of patients to further consider mental health intervention as one major component among the interventions. In addition, experimental or longitudinal studies are recommended for the screening, assessment, and psychosocial support intervention of patients with cancer.

## Data availability statement

All data generated and analyzed in this study is included in this work and available from the corresponding first authors upon reasonable request.

## Ethics statement

The studies involving human participants were reviewed and approved by Institutional Review Board, College of Medicine and Health Sciences (IRB/CMHS) at the University of Rwanda with the reference number (Ref. No: 136/UR-CMHS/SPH/2018). The patients/participants provided their written informed consent to participate in this study.

## Author contributions

SH, TM, and EB contributed to the study conceptualization, design, data acquisition, and study administration and searched the literature. SH contributed to the study administration. EB and SH drafted the manuscript, conception, data curation, analysis and interpretation of data, visualization, and validation. FK and PN contributed to the acquisition of data and conceptualization, edited the manuscript, and revised the manuscript critically for important intellectual content. SJ had a supervisory contribution to the study and substantially contributed to resource availability. All authors critically revised the manuscript and approved the final version of this manuscript.
